# Polyfunctional T cells and unique cytokine clusters imprint the anti rAAV2/rAAV9 vector immune response

**DOI:** 10.3389/fimmu.2024.1450524

**Published:** 2024-11-25

**Authors:** Stephan J. Holtkamp, Florian R. Lagoda, Adam Lister, Pradeep Harish, Ulrike Kleymann, Theresa Pesch, Chai Fen Soon, Munir Pirmohamed, Dean Naisbitt, Mark Trautwein

**Affiliations:** ^1^ Drug Discovery Sciences, Bayer AG, Wuppertal, Germany; ^2^ Department of Pharmacology and Therapeutics, Institute of Systems, Molecular and Integrative Biology, University of Liverpool, Liverpool, United Kingdom; ^3^ Wolfson Centre for Personalised Medicine, University of Liverpool, Liverpool, United Kingdom

**Keywords:** adeno-associated virus, immunogenicity, CD4 T cell immunity, gene therapy, polyfunctional T cells, T cell secretome

## Abstract

Polyfunctional T cells programmed to perform activities such as degranulation of lytic enzymes and simultaneous production of multiple cytokines are associated with more effective control of viral infections. Immune responses to recombinant adeno-associated virus (rAAV) vector delivery systems can critically influence therapeutic efficacy and safety of gene therapy. However, knowledge of polyfunctional T cells in anti-AAV immune responses is scarce. To bridge this knowledge gap, we have investigated the polyfunctionality of primary human CD4 T cells from healthy donors after *in-vitro* exposure to rAAV2 or rAAV9 vectors. By performing proliferation assays of co-cultured T cells and rAAV pulsed monocyte-derived dendritic cells from healthy donors we demonstrate T cell reactivity of 43% and 50% to rAAV2 and rAAV9 vectors, respectively. We validated this frequency in a second screen using another set of healthy donors measuring CD25 and CD71 T cell activation. Single T cell secretome analysis of reactive donors uncovered a Th1 pro-inflammatory, cytolytic and chemoattractive cytokine release profile after stimulation with rAAV2 or rAAV9 vectors. 12.4% and 9.6% of the stimulated T cells displayed a polyfunctional cytokine response, respectively, including elevated polyfunctional inflammatory indices. These responses were characterized by cytokine clusters such as Granzyme B, MIP1-α and TNF-α released in combination by single T cells. Overall, our results provide insights into adaptive immunity with rAAV vector serotypes which will be important in advancing gene therapy safety, vector selection, immunogenicity assessment and better patient selection for AAV gene therapy.

## 1 Introduction

There have been significant advances in gene therapy recently, especially with the deployment of recombinant adeno-associated viruses (rAAVs) as delivery vectors and several products winning market approval (1–3). Whilst rAAV-based vector therapies have demonstrated their prowess in efficiently transducing target tissues and maintaining long-term transgene expression (4–7), clinical translation of many candidate rAAV-mediated gene therapies remains arduous, with one of the most prominent challenges being the host immune response against the viral vector and transduced cells (8, 9).

The immune system’s intricate engagement with rAAVs encompasses both humoral [for example, pre-existing neutralizing antibodies (NAbs)], and cellular components, the priming and expansion of pre-existing antigen-specific immune cells, and/or the stimulation of naïve immune responses. While NAbs have received considerable attention for their potential to impede vector transduction and reduce therapeutic efficacy, the role of CD8 and especially CD4 T cells in the context of rAAV immunity is equally significant but less well understood (10–12).

After intravenous administration of rAAV particles, the initial contact between the vectors and the host immune system occurs in peripheral blood. Dendritic cells (DCs) such as plasmacytoid DCs are one of the most efficient phagocytic cells in blood for internalizing AAV particles (13, 14). Due to Toll-like receptor 9 (TLR9) activation-induced release of type I interferons (IFN) by plasmacytoid DCs, lymphoid conventional DCs mature and become capable of antigen presentation and T cell priming (15–17). Priming of CD4 T cells as well as the direct interaction between DCs ultimately leads to B cell mediated antibody production and CD8-cytotoxic-mediated destruction of rAAV transduced cells. The CD4 T cell response is accompanied by upregulation of co-stimulatory factors CD25 and, CD40L, as well as the release of cytokines such as tumor necrosis factor α (TNF-α) and interferon-γ (IFN-γ) (18–21).

Polyfunctionality within T cell populations has garnered substantial interest due to its potential impact on the efficiency of immune responses. Traditionally, T cell responses were categorized based on single effector functions, leading to an oversimplified understanding of the complexity underlying immune reactions. Polyfunctional T cells challenge this linear perspective by demonstrating the ability to perform multiple functions at a time, a trait often associated with superior antiviral efficacy, vaccination efficiency and patient stratification for transplantation (22–27). The multifaceted nature of polyfunctionality encompasses various cytokine secretion profiles, cytotoxic potential, and proliferation capacity. Although the role of polyfunctional T cells in anti-AAV capsid immune response remains elusive, some studies identified T cells that release multiple cytokines in response to either wild-type AAV *in-vitro* or viral vectors in *post-hoc* studies of adverse reactions during gene therapy trials (13, 28, 29). The examination of T cell cytokine release as an anti-vector response has usually been detected using either single TNF-α or IFN-γ, or small scale multiplex enzyme-linked immunosorbent spot (ELISpot) or multiparametric flow-cytometric analyses, in whole blood/peripheral blood mononuclear cells (PBMCs) (24, 28, 30–33). However, these methods have limitations with respect to T cell polyfunctionality analysis and may have led to an inadequate assessment of polyfunctional T cells and cytokine signatures in studies with rAAV vectors (11, 28, 34, 35).

Currently, in accordance to the rAAV clinical trial exclusion criteria, patients with detectable pre-existing rAAV antibodies above a predefined threshold are excluded. In addition, anti-rAAV adaptive immune responses are strongly driven by rAAV-capsid-specific cytotoxic T lymphocyte reactions. The link between the development of NAbs and T cell responses has been established but requires further investigation (9, 36–38). An exploration into early anti-rAAV capsid T cell immunogenicity, with a focus on uncovering polyfunctional T cell reactivity towards gene therapy vectors, promises to offer valuable insights into the interplay between immunogenicity and gene therapy efficacy.

In this study, we present a comprehensive analysis of polyfunctional T cells in the context of anti-rAAV2 and rAAV9 vector responses *in-vitro*. We demonstrate healthy donor T cell reactivity of 43% and 50% to rAAV2 and rAAV9 vectors, respectively. Single T cell secretome analysis of the reactive donors uncovered a Th1 pro-inflammatory, cytolytic and chemoattractive cytokine release profile after stimulation with rAAV2 or rAAV9 vectors. 12.4% and 9.6% of T cells from this population displayed a polyfunctional cytokine response, respectively, including elevated polyfunctional inflammatory indices. These responses were characterized by cytokine clusters such as Granzyme B (GrzB), Macrophage Inflammatory Protein-1 Alpha (MIP1-α) and TNF-α released in combination by single T cells. Finally, correlation analyses revealed partial correlation of cytokine release with serotype positivity.

## 2 Materials and methods

### 2.1 Human samples

Blood samples for the Bayer AG study obtained from adult subjects at CRS Clinical Research Services Management GmbH were collected in accordance with ethically approved protocols and all participants gave written concent before the study commenced. Blood samples for the University of Liverpool study were taken with approval by the local NHS Research Ethics Committee and all participants gave written informed consent before the study commenced. Additionally, Leukocyte cones were purchased from the NHSBT under local ethical approval. Donors were non-age and gender specific and pre-screened for Hepatitis B S-antigen, Hepatitis C antibody and human immunodeficiency virus 1/2 (HIV-1/2) combination The time elapsed between blood extraction and the subsequent isolation of PBMCs or serum never exceeded 1 hour. EDTA monovette tubes were utilized for PBMC extraction, while uncoated monovette tubes were employed for serum isolation.

### 2.2 Recombinant AAV vectors

Recombinant AAV2 (Vectorbiolabs #7004) and recombinant AAV9 (Vectorbiolabs #7004) were used for all experiments. The rAAV2 contains both AAV serotype 2 capsid & inverted terminal repeat (ITR), and expresses Green Fluorescent Protein (eGFP). The eGFP expression is under the control of a cytomegalovirus (CMV) promoter. The rAAV9 contains a capsid from AAV9 and ITR from AAV2 and expresses enhanced eGFP under a cytomegalovirus (CMV) promoter. Both rAAVs were stored in phosphate buffered saline (PBS)/5% glycerol at -80°C until use.

### 2.3 PBMC isolation

PBMCs were isolated according to SepMate™ density centrifugation techniques. Briefly, SepMate tubes were prepared based on the blood volume required for PBMC isolation. Ficoll Paque was carefully dispensed into SepMate tubes, and equal volumes of pre-mixed blood and PBS were gently layered on top. After centrifugation with reduced acceleration and deceleration, the plasma layer was removed to minimize platelet contamination, and the visible PBMC layers were harvested and subsequently washed with PBS. Isolated PBMCs were exposed to a red blood cell lysis buffer (Sigma-Aldrich) for 10 minutes at room temperature, followed by two careful washes. Only PBMCs exhibiting a viability exceeding 97% were selected for the isolation of CD14, CD3 (containing both CD4 and CD8 T cells), or CD4 T cells. Quality of isolated PBMCs was regularly checked by flow-cytometric analysis of CD45, CD3, CD4, CD8 and CD14 (data now shown).

### 2.4 Cellular depletion and enrichment assays

CD14 monocytes or T cells (total T cells or solely CD4 T cells) were enriched using negative isolation kits (Miltenyi) following the manufacturer’s instructions. Initially, PBMCs were subjected to staining with an anti-human AB-biotin conjugate cocktail to exclude unwanted cells. Of note, the cell of interest was not labelled. Subsequently, magnet anti-biotin streptavidin-coated beads were introduced, followed by a subsequent washing step. The labeled cells were then magnetically captured while running through a LS column (Miltenyi) and the flow through contained the cell of interest. Each individual experiment underwent purity checks, ensuring isolation purity of a minimum of 92%. Surface biomarkers including CD45, CD3, CD4, CD8, and CD14 were examined for both monocyte and T cell isolation (**Table 1**). Furthermore, for the depletion of moDCs from co-cultures, CD45, CD3, CD4, CD8, CD11c, and HLA-DQ/DR were employed to guarantee effective isolation efficiency.

**Table 1:** Used antibodies with respective conjugations (conj.) and dilutions for the flow-cytometric analyses.

Antigen	Conj.	Dilution	Clone	Catalog #	Supplier
**CD11c**	APC	1:20	3.9	301614	Biolegend
**CD134**	PE-Vio770	1:50	REA621	130-120-723	Miltenyi
**CD14**	FITC	1:50	TÜK4	130-113-705	Miltenyi
**CD25**	PE PE-eFluor610	1:100 1:50	BC96 BC96	12-0259-80 61-0259-42	Thermo Thermo
**CD3**	FITC BV711	1:100 1:100	BW264/56 UCHT1	130-113-128 300463	Miltenyi Biolegend
**CD4**	PE-Cy7 PE BV510	1:100 1:100 1:100	RPA-T4 M-T466 RPA-T4	25-0049-42 130-113-254 300546	Thermo Miltenyi Biolegend
**CD154**	BV711	1:50	24-31	310838	Biolegend
**CD45**	PE FITC	1:100 1:100	HI30 HI30	12-0459-42 11-0459-42	Thermo Thermo
**CD69**	FITC	1:100	FN50	11-0699-42	Thermo
**CD71**	BV421 BV650	1:100 1:50	CY1G4 CY1G4	334121 334115	Biolegend Biolegend
**CD8**	APC SB600 BV785	1:100 1:100 1:100	RPA-T8 RPA-T8 RPA-T8	301049 63-0088-42 301045	Biolegend Thermo Biolegend
**Granzyme B**	PE	1:50	GB11	GRB04	Thermo
**HLA-DQ/DR**	SB600	1:100	LN3	63-9956-42	Thermo
**IFN-γ**	FITC	1:150	4S.B3	11-7319-82	Thermo
**Ki67**	AF700	1:50	SolA15	56-5698-82	Thermo
**TNF-α**	eFluor-405	1:50	Mab11	48-7349-42	Thermo

The antigens are in bold to facilitate view and the start of a new row.

### 2.5 moDC culture & dendritic cell maturation

Monocyte-derived dendritic cells (moDCs) underwent culture in serum-free AIM-V, supplemented with 80 ng/ml of human recombinant granulocyte/macrophage colony-stimulating factor (GM-CSF; PeproTech) and 40 ng/ml of human recombinant IL-4 (PeproTech) for a period of 5 days, with a single medium change occurring on day 2. For thymidine incorporation assays, moDCs were cultured in complete R10 medium [RPMI-1640 supplemented with L-glutamine (Sigma), 100µg/ml penicillin (Sigma), 100 U/ml streptomycin (Sigma), 5% human Ab serum (Sigma), 25µg/ml transferrin (Sigma), and 0.6 mM 4-(2-hydroxyethyl)-1-piperazineethanesulfonic acid (HEPES; Sigma)], supplemented with 160 ng/ml each of human recombinant GM-CSF and IL-4) for 6 days and involved 2 medium washes. Monocytes were seeded at a density of 1.25 x 10^6^/ml AIM-V or 7 x 10^6^/ml R10 in 6-well plates, respectively. On the final day of culture, moDCs were harvested and exposed to 50µg/ml keyhole limpet hemocyanin (KLH; Thermofisher), 40µM nitroso-sulfamethoxazole (SMX-NO), 1x 10^4^ vg/cell rAAV2 (Vectorbiolabs), or 1x 10^5^ vg/cell rAAV9 (Vectorbiolabs) for 2 hours. In serum-free AIM-V cultures, a mix of 20ng/ml human recombinant IL-6 (Merck), 20 ng/ml human recombinant TNF-α (Peprotech), human recombinant IL-1β (Peprotech), and 1µg/ml prostaglandin E2 (PGE2; Merck) were applied to mature freshly grown moDCs (39). For R10 culture, moDCs were matured using 6.25ng/ml recombinant TNF-α and 200 ng/ml lipopolysaccharides (LPS; Thermofisher), added directly to the moDC culture for 22 hours after pulsing. Before further use, moDCs were washed extensively. In case of thymidine incorporation assays, moDCs were pre-treated with 5µg/ml of anti-Programmed death-ligand 1 (PDL1) antibody (Biolegend) for 1 hour before mixed with T cells for co-culture.

Each moDC culture, both pre- and post-maturation, underwent scrutiny to ensure a minimum of 90% CD11c expression, robust levels of HLA-DQ/DR staining, and an overall viability exceeding 85%. Additionally, to advance with experiments, heightened levels of CD80, CD83, CD86, CD40, alongside decreased levels of CD209, were deemed necessary criteria. A comparison of activation factors was facilitated by calculation of a maturation index. Similar to the stimulation index, the maturation index is the ratio of activation marker level in the treatment group to the average in the immature control group.

### 2.6 Flow cytometry

Flow cytometric analysis was employed for both immune cell culture survey and the assessment of T cell activation, proliferation, and intracellular cytokines. First, cells were washed with MACS buffer (Miltenyi), now referenced as FACS buffer, containing a 1:20 diluted Bovine Serum Albumin (BSA) stock solution (Miltenyi). Subsequently, cells were exposed to appropriate AB cocktails targeting cell surface markers (including respective isotype controls). After a 30-minute incubation at 4°C, cells were washed with FACS buffer, and LIVE/DEAD™ Fixable Near-IR (Gibco) was added at a 1:10,000 dilution. For intracellular cytokine staining, cells were fixed and permeabilized using a BD Cytofix/Cytoperm™ Fixation/Permeabilization kit (BD Biosciences) for 30 min at 4°C. Post-permeabilization, antibody cocktails targeting intracellular molecules were applied for 30 minutes at 4°C. Following another wash with FACS buffer, the cells were analyzed using an Attune Nxt Flow Cytometer (Thermo Fisher) equipped with 4 lasers, maintaining a consistent flow rate of 100µl/min and invariably recording 90% of the cell suspension. Gating of stained proteins was always done using appropriate isotype and fluorescence minus one (FMO) controls. For the generation of intracellular cytokine stain scoring, population frequencies from control conditions were subtracted from frequencies of restimulated populations. The score was then depicted in a heat map.

### 2.7 T cell activation assay

T cell activation following exposure to rAAV2 and rAAV9 vectors was assessed using a 96-well co-culture setup. Freshly prepared, antigen-loaded, and matured moDCs were combined with autologous isolated CD3 T cells, initially ensuring at least 95% T cell viability. The T cells and moDCs were incubated in a 1:10 ratio (5,000 moDCs with 50,000 T cells) for 14 days using serum-free AIM-V medium. Simultaneously, a fresh batch of autologous moDCs underwent the preparation process mentioned earlier. On day 14, co-cultured T cells were washed and transferred onto 5,000 moDCs supplemented with 100 IU/ml of recombinant human IL-2 (Gibco) for 24 hours to encourage growth of primed T cells. Supernatants were collected, and cells were stained for flow cytometric analysis, targeting CD25, CD71, CD69 or CD154 with procedures mentioned earlier.

#### 2.7.1 Ranking of donors

Donors were assessed based on T cell activation, using a reaction score ranging from 1 to 10. Each well in the co-culture, under various conditions, acted as a technical replicate. T cell activation for total CD3 T cells, CD4, CD8, and double-negative (DN) T cells was recorded within each well. Any significant difference in T cell activation between the control and restimulation categorized the well as positive. For KLH, 8 technical replicates were considered, while for rAAV2 and rAAV9, 10 technical replicates for both control and restimulation were included in the count. Five wells were seeded for background noise testing. The ratio of positive wells to the total wells (within one condition) determined the reaction score. Moreover, in instances where wells with T cells only paired with matured but empty moDCs exhibited increased T cell activation, indicative of background noise, the reaction score was adjusted downwards for each positive replicate of these background noise wells.

### 2.8 T cell proliferation assay

Another approach for assessing T cell stimulation by moDCs carrying rAAV2 and rAAV9 vectors involves evaluating cell proliferation using a moDC: CD4 T cell co-culture. The moDCs, prepared earlier, were paired with autologous CD4 T cells stained with 1µM CellTrace Violet (CTV; Thermofisher) after quality checks via flow cytometric analysis. Co-culturing these CTV^+^ CD4 T cells with moDCs for 12 days in serum-free AIM V medium allowed monitoring of CTV signal dilution as an indicator of cell division. The stimulation index, derived from the ratio of dividing cells in the treatment group to the average in the control (empty moDC), facilitated comparison of proliferation rates.

#### 2.8.1 Thymidine incorporation assay

T cell proliferation following exposure to rAAV2 and rAAV9 vectors was assessed using a 96-well co-culture setup. Freshly prepared, antigen-loaded, and matured moDCs were combined with autologous isolated CD3 T cells. The T cells and moDCs were incubated in a 1:10 ratio (20,000 moDCs with 200,000 T cells) for 14 days using R10 medium. Simultaneously, a fresh batch of autologous moDCs underwent the preparation process mentioned earlier. On day 14, co-cultured T cells were washed and transferred onto 20,000 moDCs for 48 hours. 12-16 replicates where preformed per condition, per volunteer. [3H] Thymidine (0.5 μCi/well) was added to the wells for the final 16 hours of the culture period to assess rAAV-induced t-cell proliferation. Plates were harvested using a TomTec Harvester 96 (Receptor Technologies) onto filter mats and sealed with scintillation wax and counted using a MicroBeta TriLux 1450 liquid scintillation counter (PerkinElmer). The criteria for a positive rAAV response were set as any volunteer with a minimum of one well or more above the threshold of 2 + SD above the mean of the control.

### 2.9 Cytokine release analysis

To assess cytokine release from restimulated CD4 T cells on a population level, moDC:T cell co-cultures were incubated for 14 days. On the final day, CD4 T cells were washed and combined with fresh matured moDCs for 6 hours. Harvested supernatants were stored at -80°C for subsequent analysis.

Upon analysis, supernatants were brought to room temperature and evaluated using the CodePlex platform (Bruker Cellular Analysis). Duplicate samples of 11µl of supernatant or culture medium were loaded onto the human adaptive immune chip and automatically analyzed with an Isolight instrument (Bruker Cellular Analysis). Cytokine concentrations and fold changes were determined using the IsoSpeak software (Bruker Cellular analysis).

#### 2.9.1 Single cell secretome analysis

For single-cell cytokine analysis, the IsoCode platform was employed. Initially, co-cultured cells underwent PBS wash and subsequent removal of dead cells and moDCs via magnetic bead labeling. Staining commenced with anti-human CD11c-APC (Biolegend) for 30 minutes at room temperature. Following a PBS wash, cells were exposed to basic beads (Miltenyi) for dead cell adhesion and anti-APC beads (Miltenyi) for moDC targeting for 15 minutes. Isolated cells were then processed using MS columns (Miltenyi) as per the manufacturer’s instructions and immediately assessed via flow cytometry to ensure a robust live CD4 T cell yield.

Subsequent staining of CD4 T cells with a requisite fluorescent dye for the IsoCode platform was conducted as per the manufacturer’s instructions. 30,000 cells per condition were loaded onto IsoCode chips, placed in the Isolight machine, and initiated for the assay. Following an additional 13.5-hour incubation at 37°C and CO_2_ within the machine, the Isolight platform autonomously executed standard enzyme-linked immunosorbent assay (ELISA) staining steps. Data analysis utilized the IsoSpeak Software (Bruker Cellular Analysis).

### 2.10 Serotyping

To assess total antibodies against AAV serotypes 2 and 9, an anti-AAV2 and anti-AAV9 antibody ELISA Kit from Creative Diagnostics was utilized. This involved employing precoated microwells with AAV2 or AAV9 capsid proteins for sample and control application. The captured antibodies next bound to the AAV proteins, followed by an incubation with a human IgG-specific enzyme-linked polyclonal antibody. After washing steps to remove non-specific binding, the addition of 3,3′,5,5′-tetramethylbenzidine (TMB) substrate solution led to color development proportionate to the sample’s AAV2 or AAV9 antibody concentration. The reaction was stopped with a stop solution, and absorbance at 450/620 nm was measured as per the manufacturer’s instructions for the calculations of results.

Genosafe GmbH conducted measurements of neutralizing antibody titers in donor serum using a cell-based assay. This assay evaluated the impact of anti-AAV2 or anti-AAV9 neutralizing antibodies on the transduction of HeLa cells by a recombinant AAV2 or recombinant AAV9 vector carrying the luciferase gene reporter. Signal strength decreased with rising concentrations of neutralizing factors. However, these assays did not quantitatively measure the signal inhibition down to the cut point value, employing appropriate negative and two positive controls as benchmarks.

### 2.11 Statistics

All data figures represent merged information from independent biological replicates, with any exceptions detailed in the figure legends. Each biological replicate underwent specific control experiments. Replicate validation was conducted independently, and merging occurred only when all showed consistent results. Comprehensive statistical analysis utilizing GraphPad Prism (version 10.1) incorporated both normal and non-normal data distributions, employing various tests including student’s t-test, one- and two-way ANOVA and, Mann-Whitney test; *p < 0.05, **p < 0.01, ***p < 0.001, and ****p < 0.0001 as indicators of significance as well as linear regression fit to calculate the *Pearson R square (R^2^)* value. If a *post hoc* test was employed, only the results of the *post hoc* test are shown in the figure.

## 3 Results

### 3.1 Evaluation of anti AAV vector immune responses with DC:T cell co-cultures

**Figure 1:**
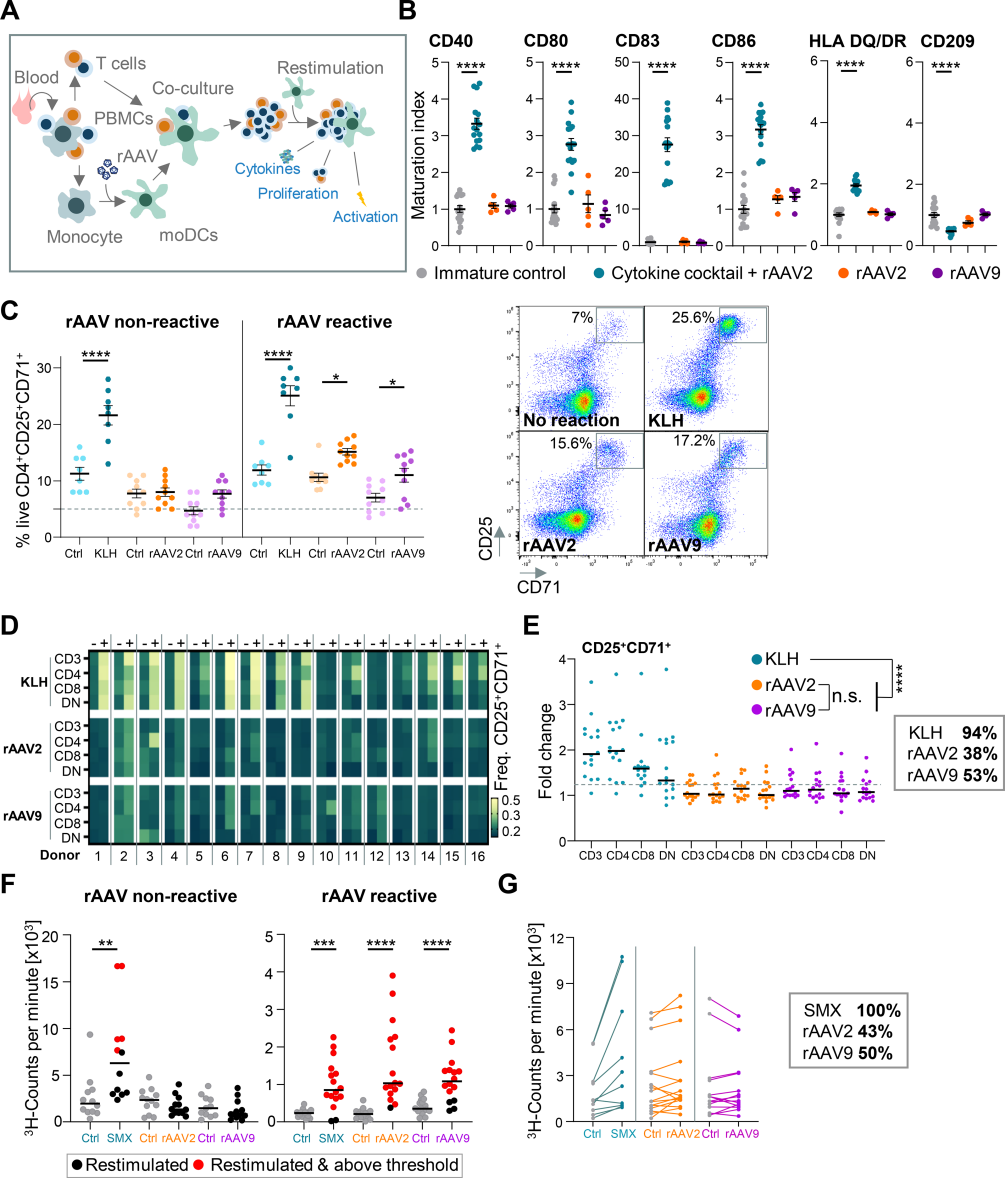
Evaluation of anti rAAV vector immune responses within DC:T cell co-cultures. **(A)** Schematic overview of immune cell isolation from fresh blood and ensuing co-culture of recombinant adeno-associated virus (rAAV) vector-pulsed monocyte-derived dendritic cells (moDCs) with autologous T cells. Cytokine release, T cell proliferation and activation are chosen readouts. **(B)** Maturation index [MFI(treated) divided by MFI(immature/untreated control)] for CD40, CD80, CD83, CD86, HLA DQ/DR and CD209 measured on the surface of moDCs with or without incubation of a cytokine cocktail and rAAV2 vectors or rAAV2/rAAV9 alone after 24 h. Data presented as mean ± SEM (n = 4-16 biological replicates), with ****p<0.0001; * = Tukey’s multiple comparisons post hoc test. **(C)** *Left:* Comparing live exemplary CD4+CD24+CD71+ frequencies between a reactive and non-reactive donor after KLH, rAAV2, or rAAV9 stimulation and controls (ctrl) after 24 h restimulation. The dashed line shows CD4+CD24+CD71+ T cells only receiving unpulsed moDCs. Data presented as mean ± SEM (n = 8-10 technical replicates), with *p<0.05; ****p<0.0001; * = Tukey’s multiple comparisons post hoc test. *Right:* Flow-cytometric analysis of CD25 and CD71 on reactive or non-reactive live CD4 T cells after 24 h restimulation with KLH, rAAV2 or rAAV9. **(D)** Overview depicting frequency of CD25+CD71+ T cells differentiated by CD3+, CD4+, CD8+ or CD4-CD8- double-negative (DN) markers after restimulation with KLH, rAAV2, rAAV9 (marked with a plus) or controls (marked by a minus) for 16 donors. **(E)** Fold change of CD25+CD71+ T cells differentiated by CD3+, CD4+, CD8+ or CD4-CD8- double-negative (DN) markers after restimulation with KLH, rAAV2, rAAV9. Dashed line marks the control value + 2xSD threshold. The box presents the frequency of reactive donors. Data presented as mean with ****p<0.0001; * = Tukey’s multiple comparisons post hoc test. **(F)** Representative rAAV-reactive and non-rAAV-reactive donor for [^3^H] thymidine proliferation assay following SMX-NO, rAAV2, rAAV9, and control(ctrl) after 48 h restimulation. rAAV and SMX-NO positive volunteers contain one well or more above the threshold of 2 x SD above the mean of the control (red dots; n = 12 technical replicates). *Data presented as median with* **p<0.01; ***p<0.001; ****p<0.0001; * *= Mann-Whitney test*. **(G)** Comparing ^3^H-counts per minute across all donors after SMX-NO, rAAV2, or rAAV9 restimulation in comparison to controls (ctrl) after 48 h restimulation. The box presents the frequency of reactive donors. Connected points mark replicates of the same donor (n = 10-16 biological replicates).

After intravenous administration, rAAVs persist in peripheral blood, encountering antigen-presenting cells (APCs) in proximity. Monocyte-derived DCs (moDCs) efficiently internalize AAV particles *in-vitro*. While AAVs typically do not undergo uncoating in DCs, they are catabolized, and capsid peptides are loaded on HLA molecules to be presented to T cells (14). Our aim was to establish an assay to scrutinize the interaction between T cells and DCs presenting rAAV vector peptides (**Figure 1A**). To achieve this, we refined a 6-day culture of CD11c^+^ HLA-DQ/DR^hi^ moDCs from primary monocytes (**Supplementary Figure S1A**). Following a 2-hour pulse with rAAV2 or rAAV9 vectors, moDCs received a maturation cocktail for 24 hours, resulting in the upregulation of typical DC maturation markers such as CD40 or CD83 and downregulation of CD209, in comparison to rAAV2 or rAAV9 vector pulse alone (**Figure 1B**, **Supplementary Figure S1B**). In patients, this maturation of immune cells is typically initiated by plasmacytoid DCs and this was mimicked *in-vitro* in our experiments using moDCs.

Next, we dissected the rAAV2- and rAAV9-induced T cell responses. To this end, autologous CD3 T cells were primed with pulsed and matured moDCs and, after 14 days, received a second set of moDCs pulsed with keyhole limpet hemocyanin [KLH; a highly immunogenic metalloprotein found in the hemolymph of the giant keyhole limpet and commonly used in immunogenicity assessments (40, 41)], rAAV2, rAAV9 vectors, or left unpulsed (referred to as control) for 24 hours. Subsequently, CD3 T cells reactive to KLH, rAAV2, or rAAV9 vectors were characterized by elevated expression of the activation markers CD25 and CD71, compared to non-rAAV-reactive donors or controls (**Supplementary Figure S1C**). Despite being a universal activation marker, a time course experiment of CD69 in addition to CD25 and CD71 allowed for the exclusion of CD69 due to a quick downregulation within 24h of restimulation (**Supplementary Figure S1D**). Further analysis revealed an increased frequency of double positive CD25+CD71+ T cells as a reliable readout characterizing reactive donors in response to KLH, rAAV2, or rAAV9 vector restimulation (*CD69 data not shown as no effect was visible after 24h*. **Figure 1C**).

To now comprehensively understand the T cell reactivity on a population level, we repeated the previously mentioned co-culture method in a screening assay to evaluate donor responses to KLH, rAAV2, or rAAV9 vectors. Screening CD4+, CD8+, or CD4-CD8- double-negative (DN) CD3 T cells from 16 donors identified a 94% reactivity to KLH, 38% reactivity to rAAV2 vectors, and 53% reactivity to rAAV9 vectors using a 1.2-fold change in threshold for CD25+CD71+ double positive T cell frequency. Interestingly, on a population level, we did not observe significant differences in the overall reactivity between T cell subtypes in the rAAV2 and rAAV9 treated groups (**Figures 1D, E**).

To complement the T cell activation analysis and further corroborate our findings, we performed a second donor screen and used T cell proliferation as readout. In this assay, autologous CD3 T cells were primed with pulsed and matured moDCs and, after 14 days, received a second set of moDCs pulsed with nitroso-sulfamethoxazole [SMX-NO; a widely used reactive metabolite of the antibiotic SMX acting highly immunogenic (42)], rAAV2 or rAAV9 vectors, or left unpulsed for 48 hours. [^3^H]Thymidine (0.5 μCi/well) was added for the last 16 hours of the culture period. Reactive donors show elevated ^3^H-counts per minute in comparison to non-reactive donors (**Figure 1F**). The t cell proliferation screen of up to 16 additional donors revealed 100% reactivity to SMX-NO, 43% reactivity to rAAV2 vectors and 50% reactivity to rAAV9 vectors demonstrating congruent results (**Figures 1G**, **Supplementary Figure S1E**).

This highlights an *in-vitro* anti-rAAV vector response marked by elevated expression of conventional T cell activation factors and enhanced proliferation observed in 38-43% and 50-53% of donors exposed to rAAV2 and rAAV9 vectors, respectively.

### 3.2 Proliferating anti-rAAV2 and anti-rAAV9 CD4 T cells produce and release cytokines within 6 hours after re-stimulation

**Figure 2:**
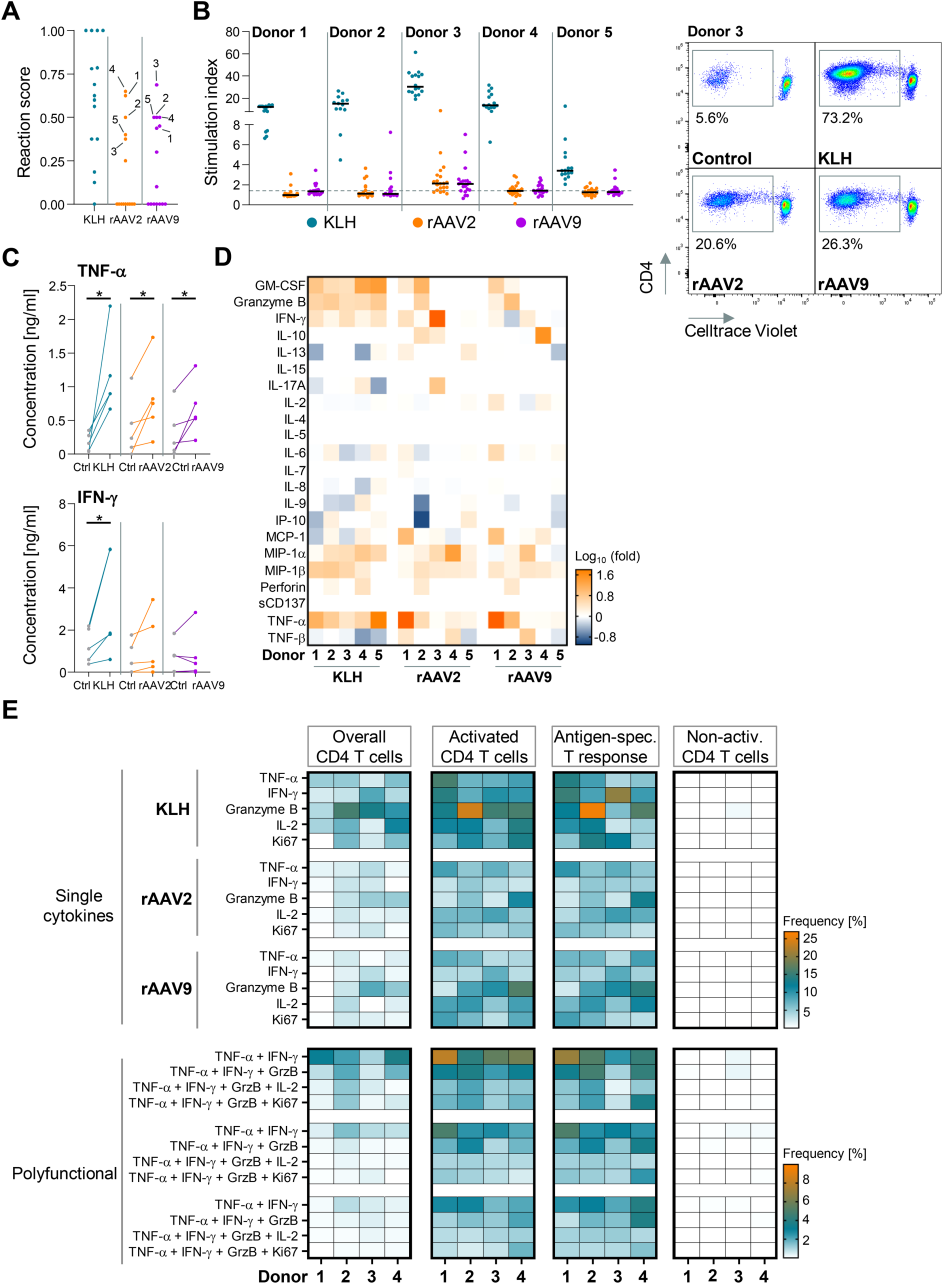
Proliferating anti-rAAV2 and anti-rAAV9 T cells produce and release cytokines within 6 h after re-stimulation. **(A)** Reaction score of KLH-, rAAV2- and rAAV9-screened donors. Five most reactive donors against rAAV2 and rAAV9 chosen for further analysis are marked by a line. **(B)** *Left:* T cell proliferation-based stimulation index (number of proliferating cells (treated) divided by number of proliferating cells (untreated control)) after 24 h restimulation of CD4 T cells with KLH-, rAAV2-vector- or rAAV9-vector-pulsed monocyte-derived dendritic cells (moDCs) in comparison to dashed line representing unstimulated controls + 2xSD. Data presented as mean (n= 10-20 technical replicates for 5 biological replicates). *Right:* Exemplary flow-cytometric analysis of CD4 and Celltrace Violet gated on live CD3 T cells after 10 days of coculture with either KLH-, rAAV2-vector-, rAAV9-vector-pulsed or empty moDCs from donor 3. **(C)** Comparing concentrations of TNF-α (*top*) or IFN-γ (*bottom*) in culture supernatant after 6 h KLH, rAAV2, or rAAV9 restimulation in comparison to controls (ctrl). Connected points mark data from the same donor. *p<0.05; * = Tukey’s multiple comparisons post hoc test (n = 5 biological replicates). **(D)** Heat map depicting the fold change of cytokines in culture supernatant after 6 h KLH, rAAV2, or rAAV9 restimulation in comparison to controls. (n = 5 biological replicates). **(E)** Heat map showing the frequency of single cytokines (*top*) or cytokine combinations (polyfunctional; *bottom*) after 6 h KLH, rAAV2, or rAAV9 restimulation after subtraction of control frequencies for overall CD4 T cells, activated CD4 T cells, T cells acting in a antigen-specific immune response (= Antigen-spec. T response) or non-activated (non-activ.) T cells (n = 4 biological replicates).

To select eligible donors for further investigation, the previously examined donors (**Figures 1D, E**) were ranked based on their cellular reactivity against rAAV2 and rAAV9 vectors. Using the integrated data from the T cell activation screen in analysed T cell subtypes, we generated a scoring system ranging from 0 (no reaction) to 1 (complete reaction across all technical replicates and investigated T cell subtypes) (**Supplementary Figure S2A**). Amongst these, donors 4, 1, 2, 5, and 3 exhibited the most pronounced T cell activation for rAAV2, while for rAAV9, donors 3, 5, 2, 4, and 1 showed the highest activation, albeit in a different order (**Figure 2A**).

To confirm the robustness of rAAV2 and rAAV9 vector reactivity and to better understand the role of T cells in the anti-rAAV vector response, we recalled the top 5 ranked donors (**Figure 2A**) for an in-depth analysis. In following analyses, we focused on CD4 T cells only due to their pivotal and central role in the anti-rAAV vector response. Subsequently, respective donors underwent fluorescence-based proliferation analysis. The priming of CellTrace Violet+ (CTV+) CD4 T cells by autologous matured moDCs for 9 days resulted in proliferation when pulsed with KLH, rAAV2, and rAAV9 vectors across all 5 donors. The stimulation index of proliferating CD4 T cells did not differ between serotypes but exhibited variability amongst donors (**Figure 2B**).

In general, anti-viral immune responses are characterized by cytokine release not only from classical cytotoxic CD8 lymphocytes but also from cytotoxic T_h_1-phenotypic CD4 T cells (43, 44). To investigate this further, we conducted bulk multiplex ELISA analysis on supernatants harvested from activated and proliferating CD4 T cells after a 6-hours restimulation. This analysis revealed a TNF-α and IFN-γ response, along with the release of MIP-1α, MIP-1β, Granzyme B, and Monocyte chemoattractant protein-1 (MCP-1) into the supernatant compared to controls (**Figures 2C, D**). Despite this, variability in cytokine release was observed amongst donors, with consistent minor differences between serotypes (**Figure 2D**).

To validate the cytokine release, T cell activation and proliferation data and discern polyfunctional T cells, we further performed multiparametric intracellular cytokine staining using flow cytometry. Four out of the five donors (donors 2-5, due to the unavailability of donor 1) were recalled. CD4 T cells were stimulated twice with autologous, pulsed, and matured moDCs. Stimulated live T cells were categorized into overall T cells (CD3+CD4+) or further subdivided into activated CD4 T cells (CD25+CD71+), antigen-specific T cell response (CD25+CD71+CD134+CD154+), or non-activated T cells (CD25-CD71-) based on surface marker expression (**Supplementary Figure S2B**). Compared to control conditions, KLH-restimulated CD4 T cells from donors 1-4 exhibited increased frequencies of CD25+CD71+, CD134+CD154+, as well as CD25+CD71+CD134+CD154+ cells. rAAV2- and rAAV9-restimulated T cells showed reduced but still discernible increases in frequencies for all four donors. CD25+CD71+ T cells showed the greatest increase in frequency overall, while CD134+CD154+ and quadruple positive T cells showed weaker increases compared to control conditions (**Supplementary Figure S2C**). Intracellular cytokine staining showed no staining in non-activated T cells, while activated and CD4 T cells clustered in the antigen-specific T cell response showed a strong signal of cytokines including TNF-α, IFN-γ, GrzB, and IL-2 as well as combinations thereof (**Figure 2E**). The extent of cytokine accumulation varied among donors both in single cytokine and polyfunctional cytokine analysis. Despite lower frequencies of double, triple, or quadruple cytokine-positive T cells compared to single cytokine-positive T cells, the frequencies of polyfunctional T cells were increased significantly after restimulation with KLH, rAAV2 and rAAV9. Polyfunctional T cells also showed partial upregulation of Ki67, indicating a proliferative state (**Figure 2E**, **Supplementary Figure S2D**).

In conclusion, restimulation ranging from 6 to 24 hours effectively induced cytokine release in pre-primed CD4 T cells, showcasing both mono- and polyfunctional attributes. These attributes signify that a Th1 pro-inflammatory, chemotactic, and cytolytic behavior is exhibited by CD4 T cells upon exposure to rAAV2 and rAAV9 vectors.

### 3.3 rAAV2 and rAAV9 vector restimulation by dendritic cells causes polyfunctional cytokine release from CD4 T cells

**Figure 3:**
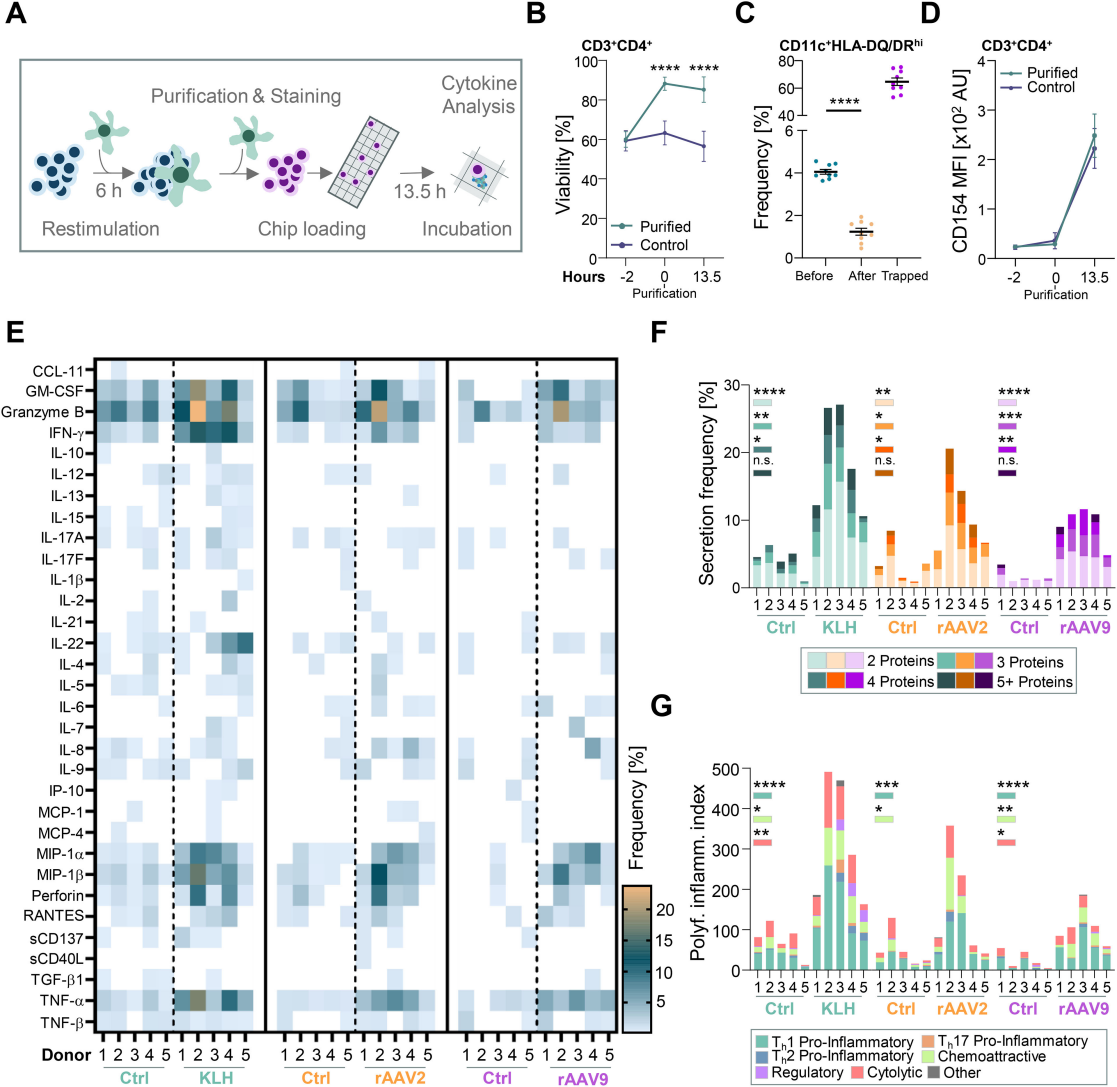
rAAV2 and rAAV9 vector restimulation by dendritic cells causes polyfunctional cytokine release from T cells. **(A)** Schematic overview of the single immune cell analysis platform and workflow including 6 h restimulation, purification, staining, chip loading, 13.5 h incubation and analysis. **(B)** Viability of CD3+CD4+ T cells before (-2 h), during (0 h) and 13.5 h after purification in comparison to control/non-purified T cells. Data presented as mean ± SEM (n = 5 biological replicates), with ****p<0.0001; * = paired student’s t-test. **(C)** Frequency of CD11c+HLA-DQ/DR^hi^ monocyte-derived dendritic cells (moDCs) before depletion, after depletion and frequency of cells in the depleted fraction (trapped). Data presented as mean ± SEM (n = 9 biological replicates), with ****p<0.0001; * = paired student’s t-test. **(D)** Mean Fluorescence Intensity (MFI) CD154 on the surface of gated live CD3+CD4+ T cells before (-2 h), during (0 h) and 13.5 h after purification in comparison to control/non-purified T cells. *Data presented as mean ± SEM (n = 10 biological replicates).* **(E)** Heat map depicting the frequency of CD4 T cells releasing certain cytokines into supernatant of Isolight chip chambers by CD4 T cells 19.5 h post KLH, rAAV2, or rAAV9 restimulation in comparison to controls (ctrl) (n = 5 biological replicates). **(F)** Calculated secretion frequency of CD4 T cells releasing 2 proteins, 3 proteins, 4 proteins or >5 proteins at a time into the supernatant of Isolight chip chambers 19.5 h post KLH, rAAV2, or rAAV9 restimulation in comparison to controls (ctrl). *p<0.05; **p<0.01; ***p<0.001; ****p<0.0001; * = Šidák correction of a two-way ANOVA test, n.s., non-significant (n = 5 biological replicates). **(G)** Calculated polyfunctional inflammation index (polyf. inflamm. index) as a sum of Th1 pro-inflammatory, Th2 pro-inflammatory, Th17 pro-inflammatory, chemoattractive, regulatory, cytolytic and other indices of CD4 T cells 19.5 h post KLH, rAAV2, or rAAV9 restimulation in comparison to controls (ctrl). *p<0.05; **p<0.01; ***p<0.001; ****p<0.0001; * = Šidák correction of a two-way ANOVA test (n = 5 biological replicates).

To further probe the role of polyfunctional T cells in anti-rAAV2 and anti-rAAV9 vector response, we sought to establish a single T cell secretome analysis platform that includes the simultaneous analysis of 32 cytokines. CD4 T cells from donors 1-5 underwent 6 hours restimulation in co-cultures using KLH, rAAV2 vector-, or rAAV9 vector-pulsed autologous moDCs. Subsequently, live CD4 T cells were enriched by eliminating dead cells and moDCs, fluorescently labeled, channeled into Isolight instrument (Bruker Cellular analysis) chip chambers, and incubated for 13.5 hours at 37°C, 5% CO_2_, to promote cytokine release (**Figure 3A**).

Throughout the entire duration of the co-culture, the viability of T cells on a population level can decline as not all T cells respond to moDC antigen presentation. To ensure assay accuracy, we optimized a workflow achieving a viability of at least 85% after depletion of dead cells, which was sustained after an additional 13.5 hours incubation (**Figure 3B**, **Supplementary Figure S3A**). Simultaneously, we depleted moDCs using a combination of anti-CD11c-APC Ab and anti-APC beads, effectively reducing the frequency of moDCs from 4% to 1% (**Figure 3C**, **Supplementary Figure S3A**). As a final quality assessment, we examined CD4 T cell activation through CD154 expression, an early activation marker known to increase in the AAV immune response (18). The isolation procedures did not alter the upregulation of CD154 on T cell surfaces immediately or after 13.5 hours following 6 hours of restimulation with rAAV2 vector-pulsed moDCs, when compared with non-enriched samples (**Figure 3D** *; rAAV9 data not shown*). This demonstrates that the procedure alone did not artificially change activation states. We employed this workflow to profile the single-cell secretome of CD4 T cells 13.5 hours after a 6-hours restimulation with rAAV2 vector-, rAAV9 vector-, or KLH-pulsed moDCs for donors 1-5, which were recalled. Analysis was conducted within a range of 480 to 1250 cells for all conditions across all donors (**Supplementary Figure S3B**). t-Distributed stochastic neighbor embedding (tSNE) analysis revealed distinct clustering of cells between control and restimulation conditions for both anti-rAAV2 and anti-rAAV9 secretomes (**Supplementary Figure S3C**).

CD4 T cells of all donors displayed a robust cytokine release upon restimulation with KLH-pulsed moDCs. Following rAAV2 and rAAV9 restimulation, CD4 T cells exhibited a similar yet reduced release of cytokines displaying Th1 pro-inflammatory, chemoattractive, and cytolytic characteristics. Across all five donors, the most prominent released cytokines included Granzyme B, TNF-α, MIP-1β, MIP-1α, IFN-γ, and GM-CSF. These signatures were characterized by substantial donor-to-donor variability but minimal differences between serotypes (**Figure 3E**).

All donors exhibited elevated frequencies of CD4 T cells releasing a minimum of two cytokines per cell, demonstrating a more polyfunctional yet donor-dependent response to anti-rAAV2 restimulation compared to anti-rAAV9 restimulation (**Figure 3F**). These findings were used to establish a polyfunctional inflammation index (PII), integrating polyfunctionality into the cytokine release data. The PII underscored a Th1 pro-inflammatory, chemoattractive, and cytolytic signature in both anti-rAAV2 and anti-rAAV9 restimulation. Notably, donor variability was pronounced in rAAV2 restimulation, particularly with donors 2 and 3 exhibiting the highest inflammation indices, whereas in rAAV9 restimulation, the PIIs were more evenly distributed, with donor 3 displaying the strongest response (**Figure 3G**).

These analyses affirm that the CD4 T cell cytokine response against rAAV2/AAV9 vectors manifests polyfunctionality characterized by a Th1 pro-inflammatory, chemoattractive, and cytolytic signature.

### 3.4 Unique cytokine clusters and combinations imprint the anti rAAV2/9 immune response

To comprehensively unravel the multi-dimensional nature of the anti-rAAV2/9 responses, we employed Polyfunctional Activity Topography Principal Component Analysis (PAT-PCA) to identify distinct cytokine clusters shared amongst donors in response to rAAV2 or rAAV9.

**Figure 4:**
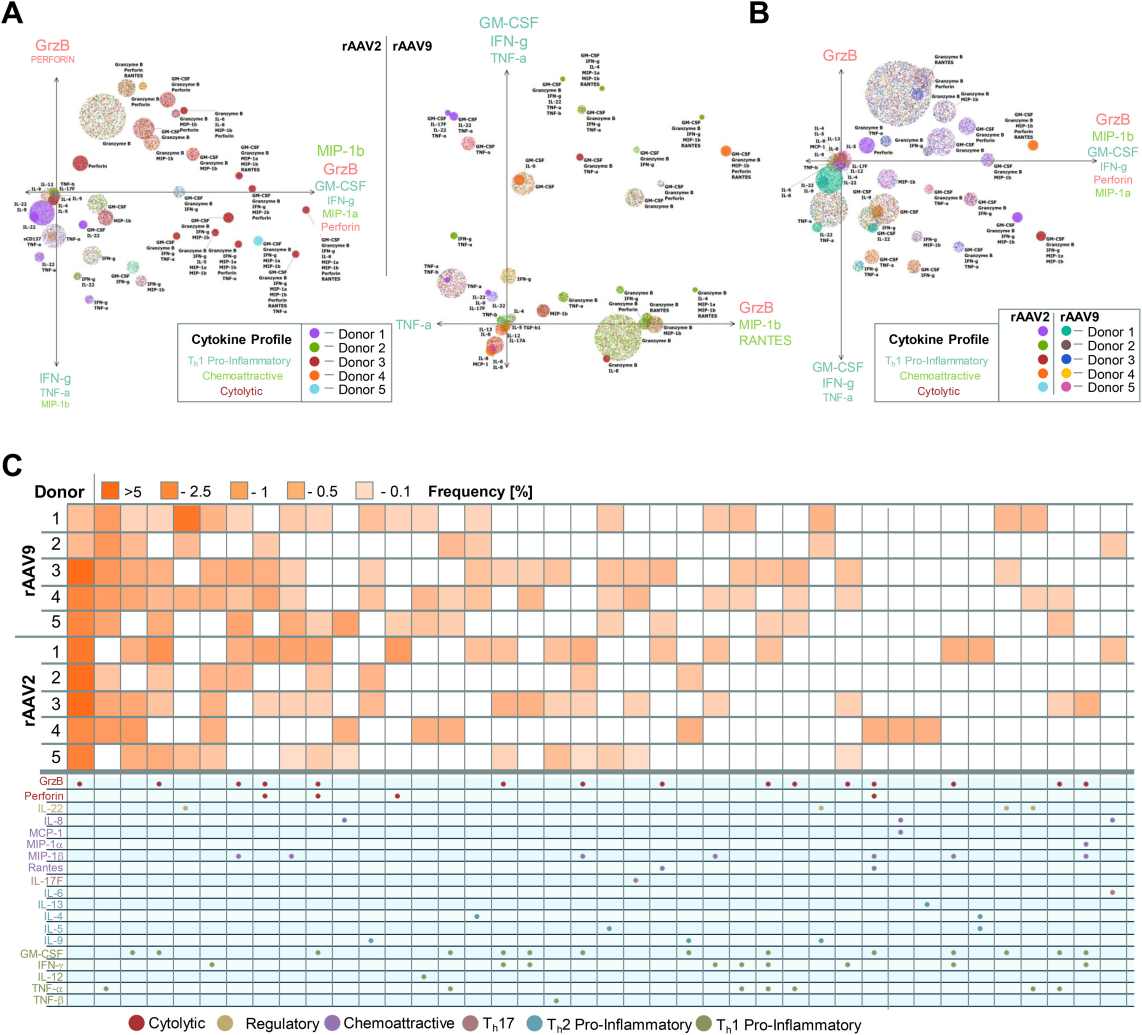
Unique cytokine clusters and combinations imprint the anti rAAV2/9 immune response. **(A)** Activity Topography principal component analysis (PAT-PCA) of cytokine clusters released by CD4 T cells after 19.5 h post restimulation with rAAV2-vector- or rAAV9-vector-pulsed monocyte-derived dendritic cells (moDCs) (n = 5 biological replicates). **(B)** Combined PAT-PCA analysis identifying shared cytokine clysters amongst rAAV2- and rAAV9-restimulated CD4 T cells (n = 5 biological replicates). **(C)** Heat map depicting the frequency of released cytokine combinations grouped by functional characteristics as cytolytic, regulatory, chemoattractive, Th17, Th2 pro-inflammatory, Th1 pro-inflammatory 19.5 h post restimulation with rAAV2 vectors and rAAV9 vectors (n = 5 biological replicates).

The CD4 T cell response against rAAV2 manifested as three components: a purely cytolytic facet, a predominantly Th1 pro-inflammatory aspect, and a third facet containing cytolytic, Th1 pro-inflammatory, and chemoattractive cytokines. Conversely, the anti-rAAV9 CD4 T cell response comprised three axes represented by TNF-α alone, a blend of Th1 pro-inflammatory cytokines, and a third axis characterized by mixed cytolytic and chemoattractive attributes. Although the cytolytic response was more pronounced in the anti-rAAV2 response compared to anti-rAAV9, the differences were relatively minor (**Figure 4A**). When integrating both rAAV2 and rAAV9 data via PAT-PCA, the cytolytic and Th1 pro-inflammatory responses appeared as distinct components, while the chemoattractive response consistently coexisted with Th1 pro-inflammatory and cytolytic molecules (**Figure 4B**). The prevalence of clusters containing multiple cytokines was relatively low compared to monofunctional release; however, polyfunctional clusters were evident across both serotypes and amongst all donors examined (**Figure 4C**).

### 3.5 The cytokine release but not T cell activation partially correlates with pre-existing immunity against AAV2 and AAV9

The anti-rAAV2 and anti-rAAV9 vector response leads to CD4 T cell activation, proliferation, and polyfunctional cytokine release. A critical criterion to exclude patients from gene therapy clinical trials focuses on pre-existing antibodies against the selected serotype. We therefore tested the hypothesis that serology aligns with the observed *in-vitro* cellular immune reaction.

**Figure 5:**
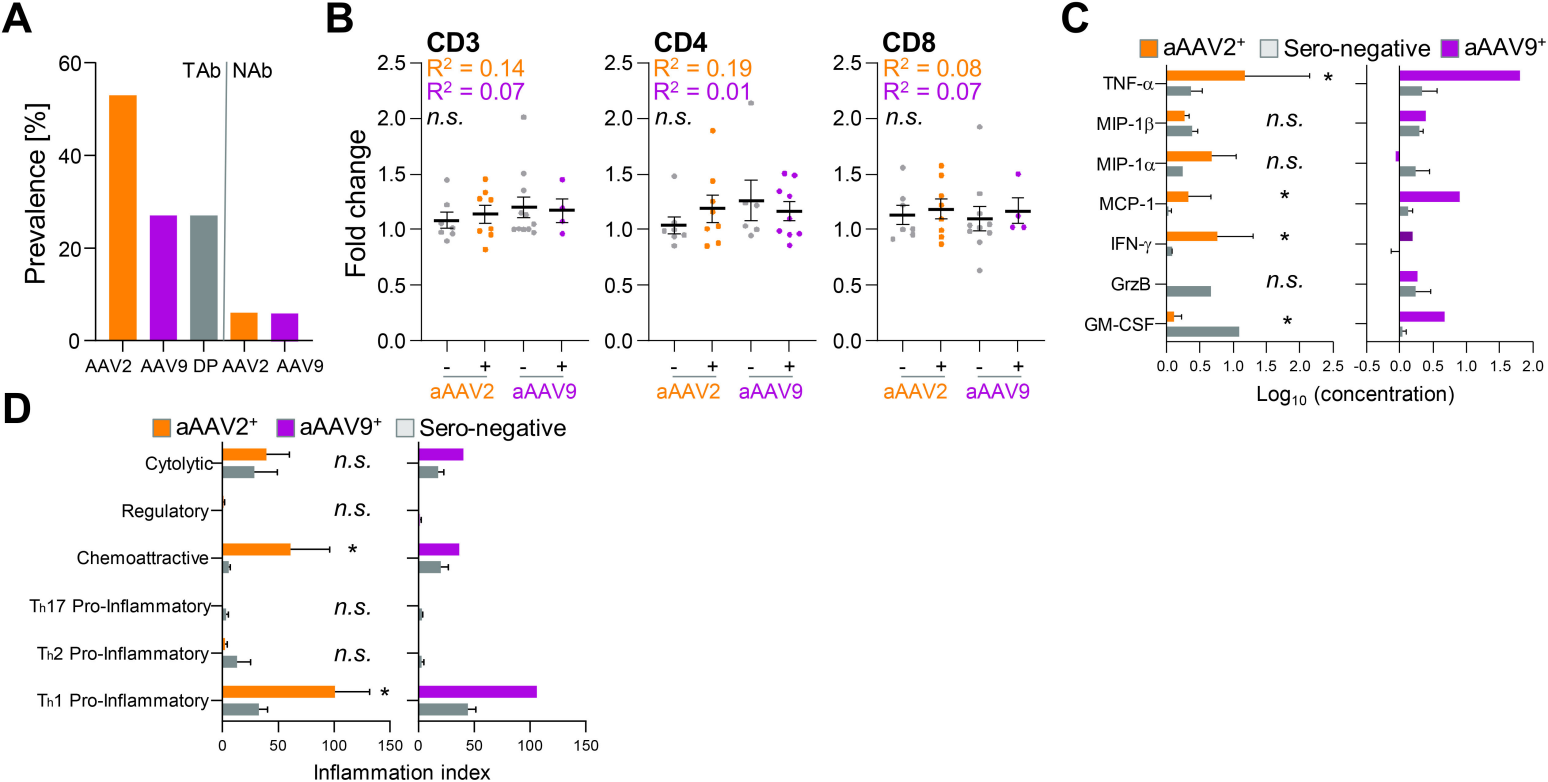
The cytokine release but not T cell activation partially correlates with pre-existing immunity against AAV2 and AAV9. **(A)** Prevalence of total antibodies (TAbs) and neutralizing antibodies (NAbs) against natural AAV2 and AAV9 or both (double positive = DP) within a cohort of 16 donors. **(B)** Fold change of CD25+CD71+ double-positive measured CD3 T cells, CD4 T cells and CD8 T cells after 24 h restimulation by rAAV2 vector (*orange*) or rAAV9 vector (*purple*) restimulation. Donors are grouped into TAb negative (minus) or TAb positive (plus) cohorts. Data presented as mean ± SEM (n = 16 biological replicates per serotype); n.s., not significant in a paired student’s t-test and *Pearson r squared (R^2^) coefficient calculated by linear regression fit*. **(C)** Concentration of released cytokines by CD4 T cells after 6 h restimulation with rAAV2 vector (*left*) or rAAV9 vector (*right*) pulsed moDCs. Donors are grouped into TAb negative (grey) or TAb positive (*orange* = aAAV2 TAb+ and *purple* = aAAV9 TAb+) cohorts (n = 5 donors for each vector type (3 donors AAV2 TAb+ and 2 donors AAV2 TAb-; 1 donor AAV9 TAb+ and 4 donors TAb-)). Data presented as mean ± SEM, with *p<0.05; * = paired student’s t-test and linear regression fit. Due to low n of aAAV9^+^ donor group, no statistics were performed **(D)** Inflammation indices of CD4 T cells 19.5 h post restimulation with rAAV2 vector (*left*) or rAAV9 vector (*right*) pulsed moDCs. Donors are grouped into TAb negative (*grey*) or TAb positive (*orange* = aAAV2 TAb+ and *purple* = AAV9 TAb+) cohorts (n = 5 donors for each vector type (3 donors AAV2 TAb+ and 2 donors AAV2 TAb-; 1 donor AAV9 TAb+ and 4 donors TAb- 4)). Data presented as mean ± SEM, with *p<0.05; * = paired student’s t-test and linear regression fit. Due to low n of aAAV9^+^ donor group, no statistics were performed.

Sera from donors 1-16 underwent testing for pre-existing antibodies — total and neutralizing — against AAV2 and AAV9 serotypes. Of the analyzed sera, 53% contained total antibodies (TAbs) against AAV2, while 27% harbored TAbs against AAV9, in alignment with reference populations (**Figure 5A**) (45). Intriguingly, all AAV9 TAb-positive sera also exhibited AAV2 TAbs. Surprisingly, only one donor exhibited NAbs against both AAV2 and AAV9 serotypes, prompting the exclusion of NAb serologies from the correlation analysis.

Comparing sero-positive versus sero-negative donors for T cell activation revealed no correlation. The fold change in CD25+CD71+ double positive CD3 T cells, irrespective whether they were CD4 or CD8 T cells, did not differ between donors positive or negative for AAV2 and AAV9 serology (**Figure 5B**). However, cytokine release was elevated in donors with TAbs against AAV2 and AAV9 (**Figure 5C**). Notably, TNF-α, MIP-1α, and IFN-γ levels were higher, while GM-CSF levels were lower, in donors positive for anti-AAV2 TAbs. Additionally, the donor with anti-AAV9 TAbs exhibited increased TNF-α, MCP-1, and GM-CSF compared to sero-negative donors.

Further exploration of cytokine versus serology correlation using the PII revealed a more pronounced chemoattractive anti-rAAV2 CD4 T cell response and Th1 pro-inflammatory response against anti-rAAV2 and anti-rAAV9 vectors in TAb-positive donors (**Figure 5D**). It is important to note that the number of correlated anti-AAV9 TAb-positive donors (and consequently also statistical analysis) was limited to one donor. However, this suggests a potential correlation between the intensity of cellular T cell responses against rAAV2 and rAAV9 vectors and pre-existing immunity indicated by TAbs.

## 4 Discussion

For the first time, on the single immune cell level we show, *in-vitro*, the involvement of polyfunctional CD4 T cell cytokine release in response to rAAV vector treatment. The CD4 T cell immune response against rAAV2 an rAAV9 vectors was marked by an upregulation of T cell activation markers CD25, CD71, CD134 and CD154, proliferative behavior and a Th1-proinflammatory, chemoattractive and cytolytic cytokine release.

Gene therapies present unique challenges to immunogenicity assessment. Immune responses to gene therapy products may compromise efficacy and patient safety. Currently, inclusion criteria for AAV gene therapy often address the patient’s antibody status. These criteria are reflected in certain threshold levels (ranging e.g. from 1:5 to 1:50 NAbs titers) but do not include cellular immunity (46–48). However, regulatory guidelines imply that cellular immunogenicity (beyond CD8 T cells) should be assayed throughout development and carefully looked after during and after therapy, for which our results offer guidance to better monitor CD4 T cells (49, 50). Our presented CD4 T cell activation profile is in alignment with previous studies in which immunizing mice with recombinant adenovirus (Ad)- influenza virus hemagglutinin (HA) but also rAAV-HA vectors was correlated with an upregulation of the activation marker CD69 on the surface of CD4 T cells in response to rAAV with anti-rAAV AB development and reactivity against rAAVs (51). T helper cell-dependent antibody formation, Th1 CD4 T cell cytokine release and CD4 T cell activation has been described in anti-rAAV immune responses in several studies (52–54) aligning with the behavior of CD4 T cells recorded in our assays. Nonetheless, most data published to date have focused on the cytotoxic CD8 T cell anti-transgene response (11, 18, 55–57). Although our initial screen did also show CD8 T cell activation, CD8 T cell cytokine release was not addressed in this study. While cytotoxic CD8 T cells are one of the most likely causes of organ damage upon systemic rAAV gene transfer, these cells rely on the interaction with CD4 T cells interacting with plasmacytoid DCs and conventional DCs to subsequently activate CD8 T cells (15, 58). This dependency is essential during naïve CD8 T cell responses and irrespective of memory CD8 T cell responses (59). Herein, the CD4 T cells were not further differentiated into memory and naïve CD4 T cell responses. Although both naïve and memory CD4 T cells play a role in *in-vitro* stimulation of T cells with rAAV vectors, a better understanding of the degree of naïve and memory as well as the inflammatory profile is needed (21). Results presented here not only complement the current findings, but also extend the understanding of anti-rAAV2 and -rAAV9 vector CD4 T cell immune responses and promote the polyfunctionality of CD4 T cells as a new criterion for the *post-hoc* and pre-therapy analysis of gene therapy products.

Polyfunctional cytokine release by CD4 T cells in response to rAAV2 and rAAV9 encompassed Granzyme B, TNF-α, MIP-1β, MIP-1α, IFN-γ, IL-2 and GM-CSF as well as combinations thereof. Several *in-vitro* studies have shown a similar single cytokine release profile measured by flow-cytometry or ELISpot assays. The comparability of study design is limited as in most studies whole PBMCs were treated only once with whole capsid or viral protein (VP) peptides instead of complex co-cultures including restimulations. Nonetheless, in a study by Bing and colleagues, PBMCs were stimulated with AAV9 viral protein peptides (previously identified to strongly bind HLA class II molecules) causing intracellular upregulation of IL-2, IFN-γ and TNF-α in CD4 T cells matching our intracellular cytokine data (60). In another study by Kuranda et al., PBMC stimulation with empty rAAV2 capsid particles led to the emergence of GrzB+ naive and memory CD4 T cells at low frequencies (28). More precise formats will be necessary in the future to better understand the exact role of CD4 T cells, but our results offer translatability using existing methods such as ELISpot or ELISA in clinical trials and immunogenicity assessments.

In this study, the number of anti-AAV2 and anti-AAV9 NAb-positive donors was considerably lower when comparing with other studies. To date, conditions for NAb prevalence assays vary widely in both preclinical and clinical settings. For instance, in Biomarin’s hemophilia A trial (NCT02576795), patients without neutralizing antibodies against AAV5 were enrolled, with a NAb assay using 25,000 vector genome-containing particles per cell (61). Uniqure used a GFP-based assay for their hemophilia B trial (NCT02396342) without reporting the multiplicity of infection (MOI), while another hemophilia B trial (NCT03489291) used a highly sensitive luciferase assay with an MOI of 378.4 (62). These differing MOIs can yield dramatically different NAb titers, even if other assay conditions are identical. Factors such as vector purity, the presence of inactive viral particles, and inhibition by other serum factors further complicate NAb assays and suggest the need for a standardized reporting unit and protocols (63).

Several studies have shown no or only partial correlation between pre-existing anti-AAV T cell responses and AAV NAb titers (64, 65). Newer approaches described correlations between for example the proportion of rAAV8-specific CD8+ T cells with a CD45RA+ CCR7− terminally-differentiated effector memory (TEMRA) cells and IFN-γ ELISpot positive responses in healthy human donors (32). Capsid-reactive T cells can be detected in a larger number of individuals in splenocytes compared with PBMCs, suggesting that rAAV-specific T cells might fail to recirculate in peripheral blood, and preferentially home to lymphoid organs offering an explanation for the missing or weak correlation identified here (66).

This study’s chief limitation is the relatively small number of individuals studied but mitigated by the fact that there was extensive investigation of each individual. Extensive analyses such as ours used in this study are needed for an in-depth evaluation in patients who develop immune responses that lead to tissue injury, for example liver injury, after gene therapy, so that we can effectively treat and prevent such reactions in the future. Specifically, our cytokine release signature can be recapitulated in clinical studies evaluating the involvement of CD4 T cell responses in such injuries or before administration in immunogenicity assessments. An increased understanding of the nature of (pre-existing) immune responses to natural or recombinant AAV infections may allow for more directed patient selection and/or interventions for the prevention or mitigation of immune responses during rAAV gene therapy. In the future, the polyfunctionality of CD8 T cells, regulatory T cells and, due to the emerge of tissue-specific capsids, relevant immune microenvironments must be defined and better understood (67–69). Our data offer valuable information about rAAV vector immunogenicity and polyfunctional CD4 T cell responses potentially aiding in the development of pre-clinical gene therapy candidates and the assessment of patient immunity during and before therapy.

## Data Availability

The original contributions presented in the study are included in the article/**Supplementary Material**. Further inquiries can be directed to the corresponding author.
