# Supplementary material for: Polyfunctional T cells and unique cytokine clusters imprint the anti rAAV2/rAAV9 vector immune response
Source: Front Immunol. 2024 Nov 25;15:1450524. doi: 10.3389/fimmu.2024.1450524 (PMC11625739; doi:10.3389/fimmu.2024.1450524)
Supplement: Supplementary file 1 [file DataSheet1.pdf]

Supplementary Material

Suppl. Figure 1: Immune cell gating and T cell activation & proliferation analysis

(A) Exemplary flow-cytometric gating of live immature and matured CD11c+HLA-DQ/DR$^{hi/intermediate}$ monocyte-derived dendritic cells (moDCs). **(B)** Histograms of CD40, CD209, CD83, CD80 and CD86 fluorescence intensity on immature and matured moDCs in comparison to isotype controls. **(C)** CD71 and CD25 histograms of KLH-restimulated and control T cells. T cells are stratified as live CD3+ T cells and further gated as CD4+, CD8+ or CD4-CD8- double-negative T cells. **(D)** CD3+ T cell activation time course experiment for CD69, CD25 and CD71 upregulation on CD3 T cells after restimulation with KLH-pulsed moDCs or respective controls for 6-48 hours *Data presented as mean ± SEM (n = 5 biological replicates)*. **(E)** ^{3}H-counts per minute of a [^{3}H] thymidine proliferation assay 48 h SMX-NO, rAAV2, or rAAV9 restimulation in comparison to respective controls (ctrl). rAAV and SMX-NO positive donors contain one well or more above the threshold of 2 x SD above the mean of the control (*red dots*). *Data presented as mean (n = 12-16 technical replicates per donor; n = 16 biological replicates)*.

Suppl. Figure 2: T cell cytokine analysis

(A) Exemplary CD25+CD71+ T cell reaction heatmap, classified as total T cells (CD3+), CD4+, CD8+ or CR4-CD8- double-negative (DN), of a donor reactive to KLH-, rAAV2- and rAAV9-mediated restimulation used for reaction score calculation. One square resembles one technical replicate of a 96 well with *blue* presenting a significant reaction over threshold and *white* no reaction. **(B)** Exemplary flow-cytometric gating of overall live CD3+CD4+, activated (CD25+CD71+), non-activated (CD25-CD71-) and antigen-specific (CD25+CD71+CD134+CD154+) T cells. **(C)** Frequency of live CD4 T cells classified as CD25+CD71+, CD134+CD154+ or CD25+CD71+CD134+CD154+ for donor 1-4. *Orange* dots resemble technical replicates of 24 h KLH-, rAAV2- or rAAV9-restimulated T cells in comparison to *green* dots presenting restimulation with unpulsed monocyte-derived dendritic cells (moDCs; control). *Data presented as mean ± SEM (n = 10 technical replicates for 4 biological replicates), with **p<0.01; ***p<0.001; ****p<0.0001; * = Šidák correction of a two-way ANOVA test*. **(D)** Exemplary flow-cytometric histoplots and pseudoplots showing the intracellular staining for TNF-α, IFN-γ, granzyme B (GrzB), IL-2 and Ki67 in live CD3+CD4+ T cells and activated CD4 T cells after 24 h restimulation with rAAV2-pulsed or unpulsed (ctrl) moDCs. *Left:* Single analyte histoplots of CD4 T cells depicting frequencies of positive cells based on isotype staining. *Right:* For polyfunctionality analysis, TNF-α^{hi}IFN-γ^{hi} live CD4 T cells are further divided into Grzb^{hi} or Grzb^{hi}IL-2^{hi} cells. Grzb^{hi}TNF-α^{hi}IFN-γ^{hi} CD4 T cells are further stained for Ki67.

Suppl. Figure 3: Single-cell cytokine analysis of CD4 T cells

(A) Exemplary gating strategy to measure the frequency of CD11c+HLA-DQ/DR^{hi} monocyte-derived dendritic cells (moDCs) and overall viability of cells before (*top*) and after (*bottom*) depletion. **(B)** Cell counts of live CD4 T cells taken for analysis in Isolight machine cell chamber chips for 5 donors *(n = 6 technical replicates)*. **(C)** 3-dimensional exemplary t-distributed stochastic neighbor embedding (T-SNE) plots of CD4 T cells after 19.5 h restimulation with rAAV2 or rAAV9 vectors or controls (Ctrl).

Fig. S1.

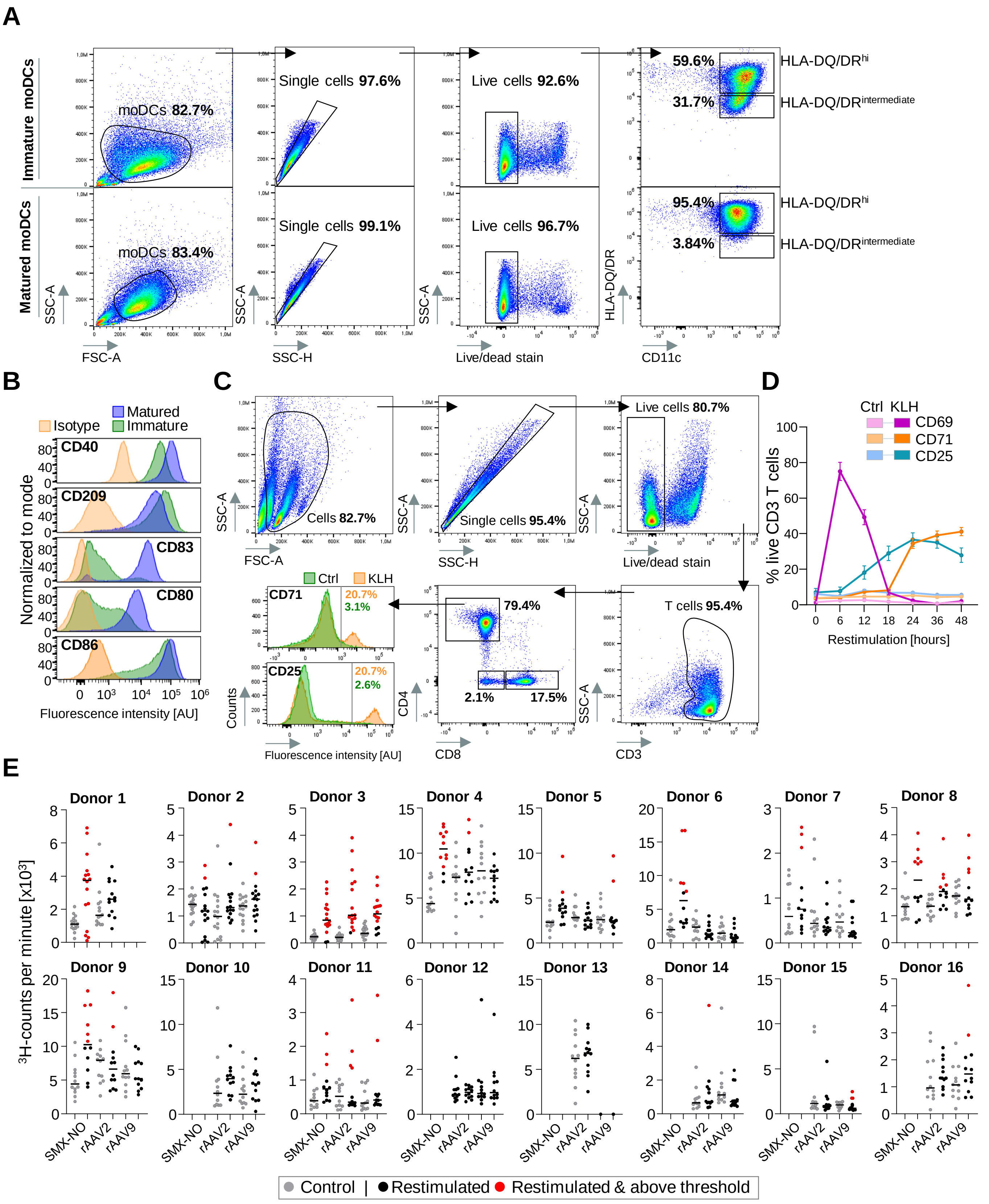

A
Immature moDCs
Matured moDCs
moDCs 82.7%
moDCs 83.4%
Single cells 97.6%
Single cells 99.1%
Live cells 92.6%
Live cells 96.7%
59.6%
31.7%
95.4%
3.84%
HLA-DQ/DRhi
HLA-DQ/DRintermediate
SSC-A
FSC-A
SSC-H
Live/dead stain
HLA-DQ/DR
CD11c
B
Matured
Isotype
Immature
CD40
CD209
CD83
CD80
CD86
Normalized to mode
Fluorescence intensity [AU]
C
Cells 82.7%
Single cells 95.4%
Live cells 80.7%
T cells 95.4%
79.4%
2.1%
17.5%
Ctrl
KLH
CD71
CD25
20.7%
3.1%
2.6%
Counts
Fluorescence intensity [AU]
CD4
CD8
CD3
D
Ctrl KLH
CD69
CD71
CD25
% live CD3 T cells
Restimulation [hours]
E
Donor 1
Donor 2
Donor 3
Donor 4
Donor 5
Donor 6
Donor 7
Donor 8
Donor 9
Donor 10
Donor 11
Donor 12
Donor 13
Donor 14
Donor 15
Donor 16
3H-counts per minute [x10³]
SMX-NO
rAAV2
rAAV9
Control | Restimulated Restimulated & above threshold

Fig. S2.

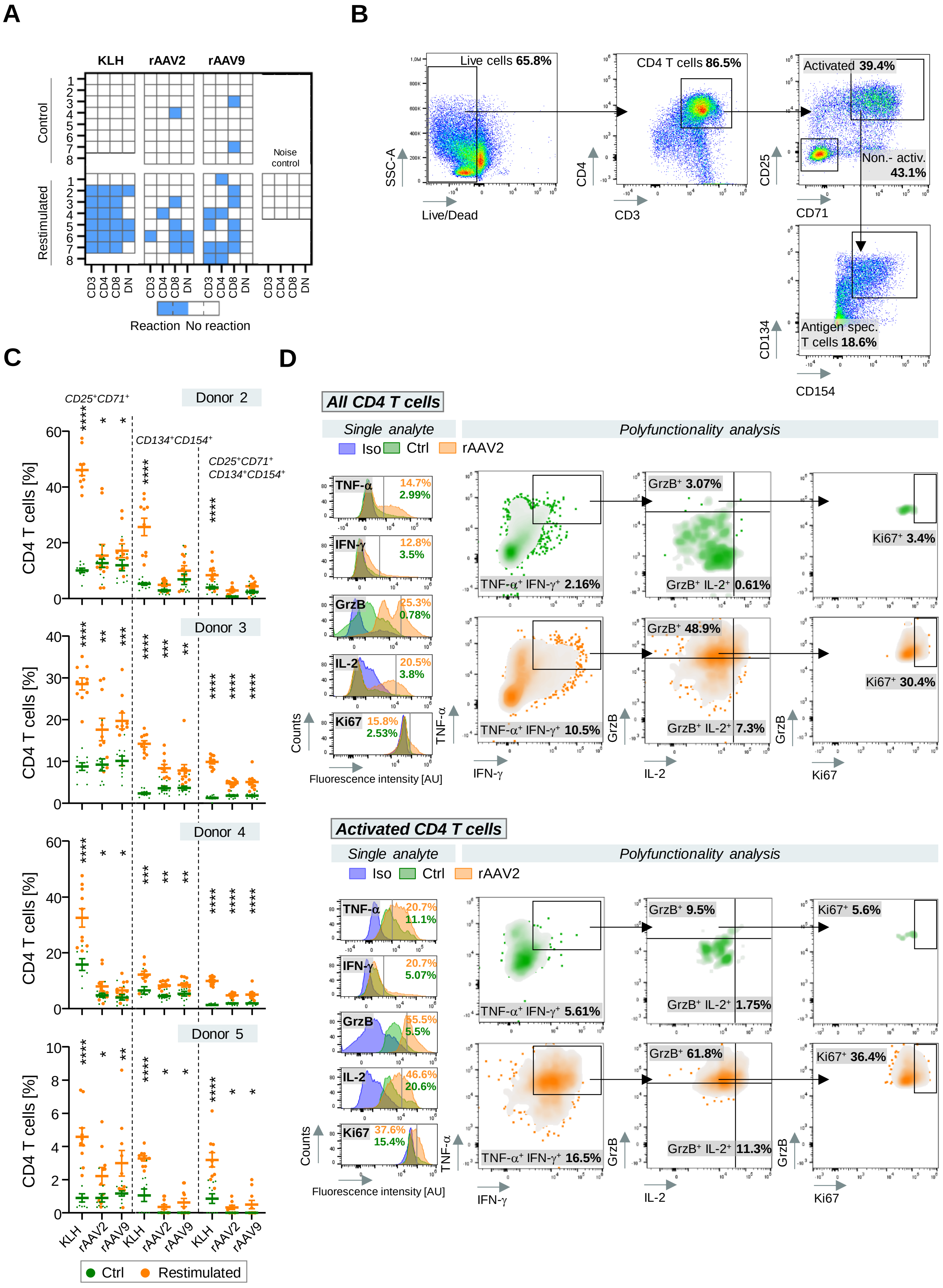
A
KLH
rAAV2
rAAV9
Control
Restimulated
Noise control
CD3
CD4
CD8
DN
Reaction No reaction
B
Live cells 65.8%
CD4 T cells 86.5%
Activated 39.4%
Non.- activ. 43.1%
Antigen spec. T cells 18.6%
SSC-A
Live/Dead
CD4
CD3
CD25
CD71
CD134
CD154
C
CD25+CD71+
CD134+CD154+
CD25+CD71+ CD134+CD154+
Donor 2
Donor 3
Donor 4
Donor 5
CD4 T cells [%]
KLH
rAAV2
rAAV9
Ctrl
Restimulated
D
All CD4 T cells
Activated CD4 T cells
Single analyte
Polyfunctionality analysis
Iso
Ctrl
rAAV2
TNF-α
IFN-γ
GrzB
IL-2
Ki67
Counts
Fluorescence intensity [AU]
TNF-α+ IFN-γ+ 2.16%
TNF-α+ IFN-γ+ 10.5%
GrzB+ 3.07%
GrzB+ 48.9%
GrzB+ IL-2+ 0.61%
GrzB+ IL-2+ 7.3%
Ki67+ 3.4%
Ki67+ 30.4%
TNF-α+ IFN-γ+ 5.61%
TNF-α+ IFN-γ+ 16.5%
GrzB+ 9.5%
GrzB+ 61.8%
GrzB+ IL-2+ 1.75%
GrzB+ IL-2+ 11.3%
Ki67+ 5.6%
Ki67+ 36.4%

Fig. S3.

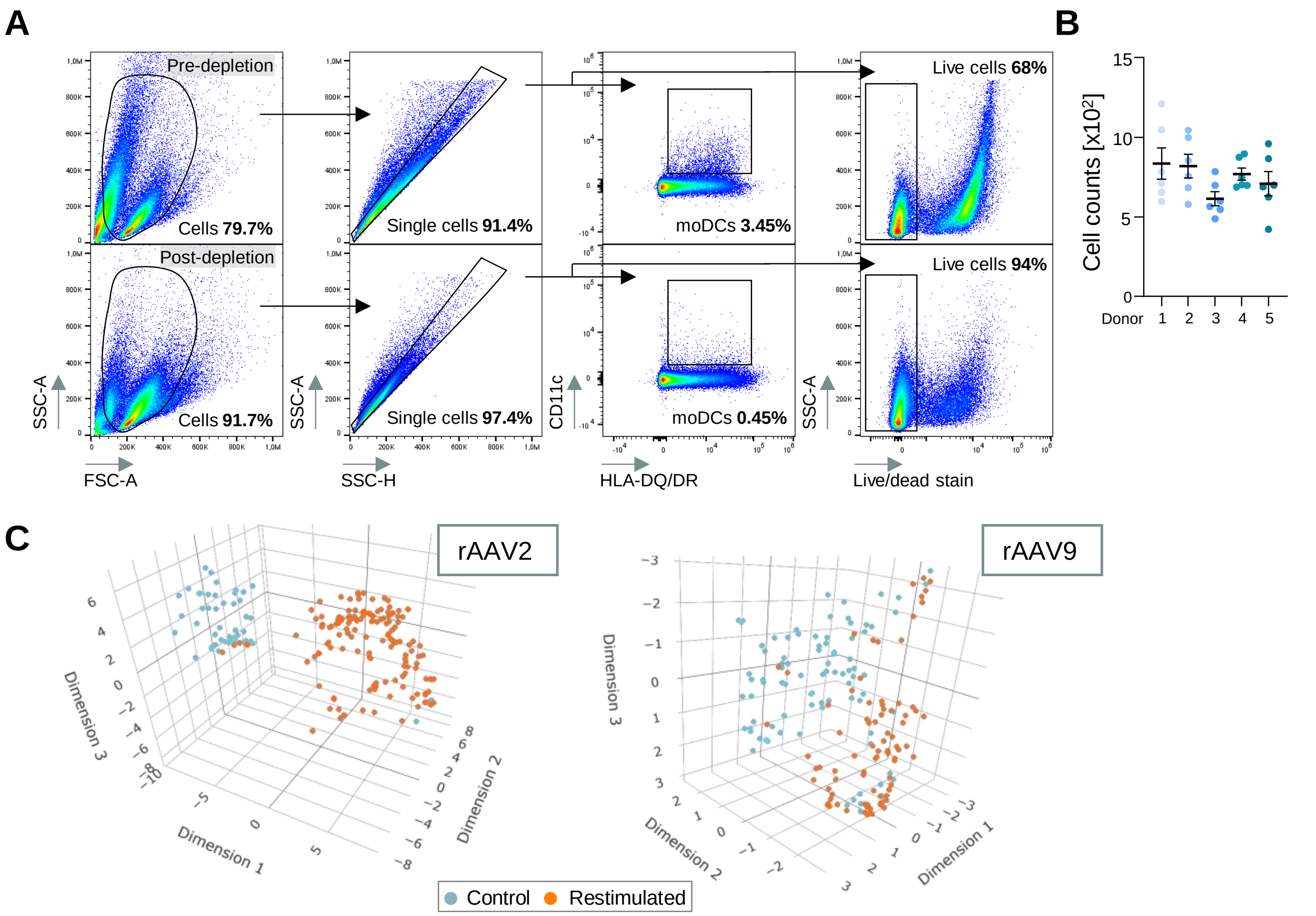
A
Pre-depletion
Cells 79.7%
Post-depletion
Cells 91.7%
SSC-A
FSC-A
Single cells 91.4%
Single cells 97.4%
SSC-H
moDCs 3.45%
moDCs 0.45%
CD11c
HLA-DQ/DR
Live cells 68%
Live cells 94%
Live/dead stain
B
Cell counts [x10²]
Donor 1 2 3 4 5
C
rAAV2
rAAV9
Dimension 1
Dimension 2
Dimension 3
Control
Restimulated
